# Supplementary material for: Structural basis for xenobiotic extrusion by eukaryotic MATE transporter
Source: Nat Commun. 2017 Nov 21;8:1633. doi: 10.1038/s41467-017-01541-0 (PMC5696359; doi:10.1038/s41467-017-01541-0)
Supplement: Supplementary file 1 — Supplementary Information [file 41467_2017_1541_MOESM1_ESM.pdf]

a

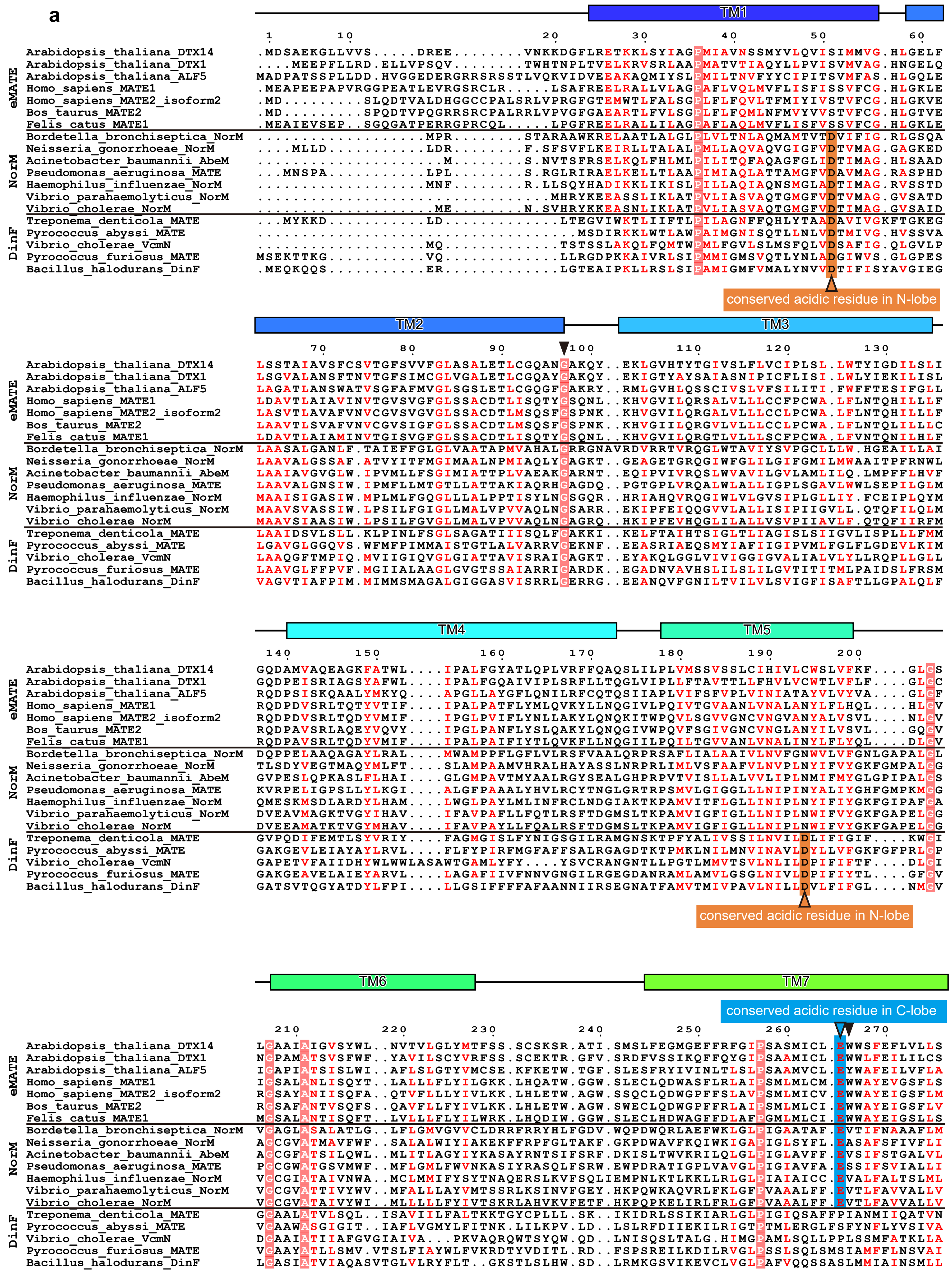
TM1
TM2
TM3
TM4
TM5
TM6
TM7
eMATE
NorM
DinF
Arabidopsis_thaliana_DTX14
Arabidopsis_thaliana_DTX1
Arabidopsis_thaliana_ALF5
Homo_sapiens_MATE1
Homo_sapiens_MATE2_isoform2
Bos_taurus_MATE2
Felis_catus_MATE1
Bordetella_bronchiseptica_NorM
Neisseria_gonorrhoeae_NorM
Acinetobacter_baumannii_AbeM
Pseudomonas_aeruginosa_MATE
Haemophilus_influenzae_NorM
Vibrio_parahaemolyticus_NorM
Vibrio_cholerae_NorM
Treponema_denticola_MATE
Pyrococcus_abyssi_MATE
Vibrio_cholerae_VcmN
Pyrococcus_furiosus_MATE
Bacillus_halodurans_DinF
conserved acidic residue in N-lobe
conserved acidic residue in N-lobe
conserved acidic residue in C-lobe

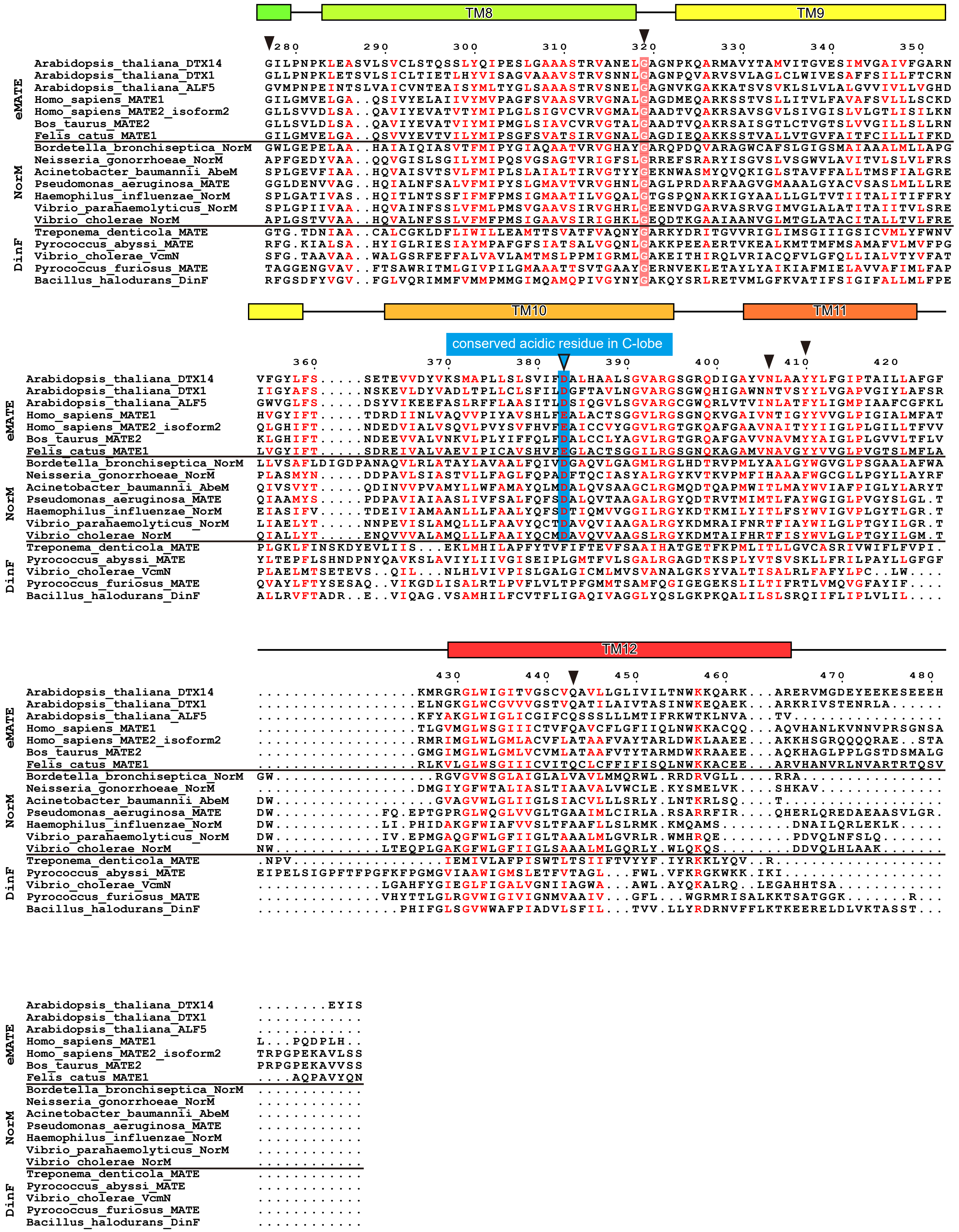

TM8
TM9
TM10
TM11
TM12
conserved acidic residue in C-lobe
eMATE
NorM
DinF
Arabidopsis_thaliana_DTX14
Arabidopsis_thaliana_DTX1
Arabidopsis_thaliana_ALF5
Homo_sapiens_MATE1
Homo_sapiens_MATE2_isoform2
Bos_taurus_MATE2
Felis_catus_MATE1
Bordetella_bronchiseptica_NorM
Neisseria_gonorrhoeae_NorM
Acinetobacter_baumannii_AbeM
Pseudomonas_aeruginosa_MATE
Haemophilus_influenzae_NorM
Vibrio_parahaemolyticus_NorM
Vibrio_cholerae_NorM
Treponema_denticola_MATE
Pyrococcus_abyssi_MATE
Vibrio_cholerae_VcmN
Pyrococcus_furiosus_MATE
Bacillus_halodurans_DinF

b

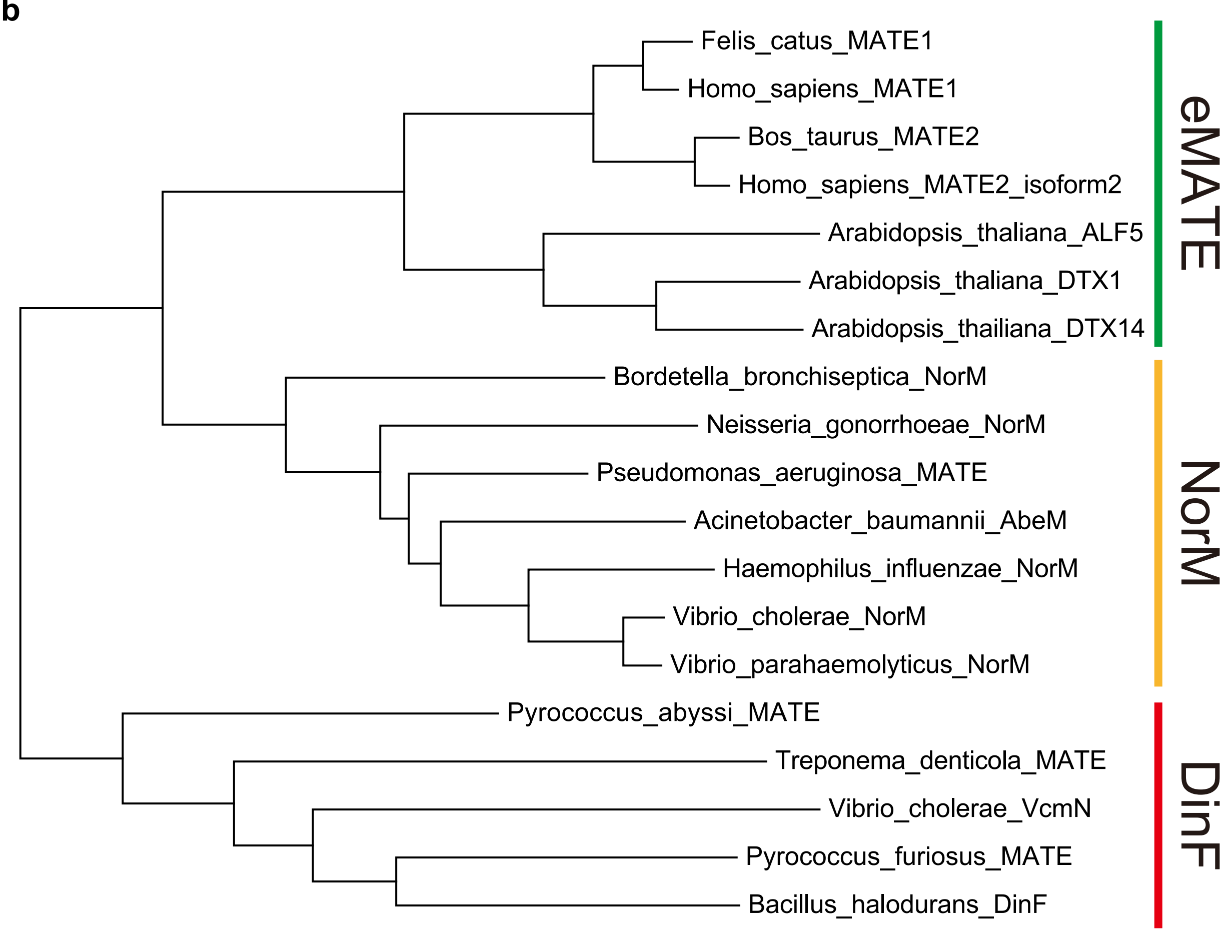

Supplementary Figure 1. Amino acid sequence alignment of the MATE transporters

(a) Amino acid sequence alignment of AtDTX14 (UniProt ID: Q9C994) and other MATE homologues, Arabidopsis thaliana DTX1 (UniProt ID: Q9SIA5), Homo sapiens MATE2 isoform2 (UniProt ID: Q86VL8-3), Homo sapiens MATE1 (UniProt ID: Q96FL8), Bos taurus MATE2 (UniProt ID: E1BIJ2), Felis catus MATE1 (UniProt ID: M3WFX8), Vibrio parahaemolyticus NorM (UniProt ID: O82855), Haemophilus influenzae NorM (UniProt ID: Q4QL47), Vibrio cholerae NorM (UniProt ID: C3LML9), Acinetobacter baumannii AbeM (UniProt ID: D0CAR7), Pseudomonas aeruginosa MATE (UniProt ID: A0A1D5BPX0), Bordetella bronchiseptica NorM (UniProt ID: Q7WJR0), Neisseria gonorrhoeae NorM (UniProt ID: D6H990), Pyrococcus furiosus MATE (UniProt ID: Q8U2X0), Pyrococcus abyssi MATE (UniProt IF: Q9V1R1), Treponema denticola MATE (UniProt ID: M2BXS7), Vibrio cholerae VcmN (UniProt ID: C3LWQ2), and Bacillus halodurans DinF (UniProt ID: Q9KAX3). The essential residues for the transport activities of the DinF and NorM subfamilies are highlighted in orange, and those for eMATE and NorM are highlighted in sky-blue. The conserved residues discussed in the main text are indicated by black triangles. (b) Phylogenetic tree of MATE homologues, based on the sequence alignment in panel (a). The phylogenetic tree was generated with the program MEGA7[1].

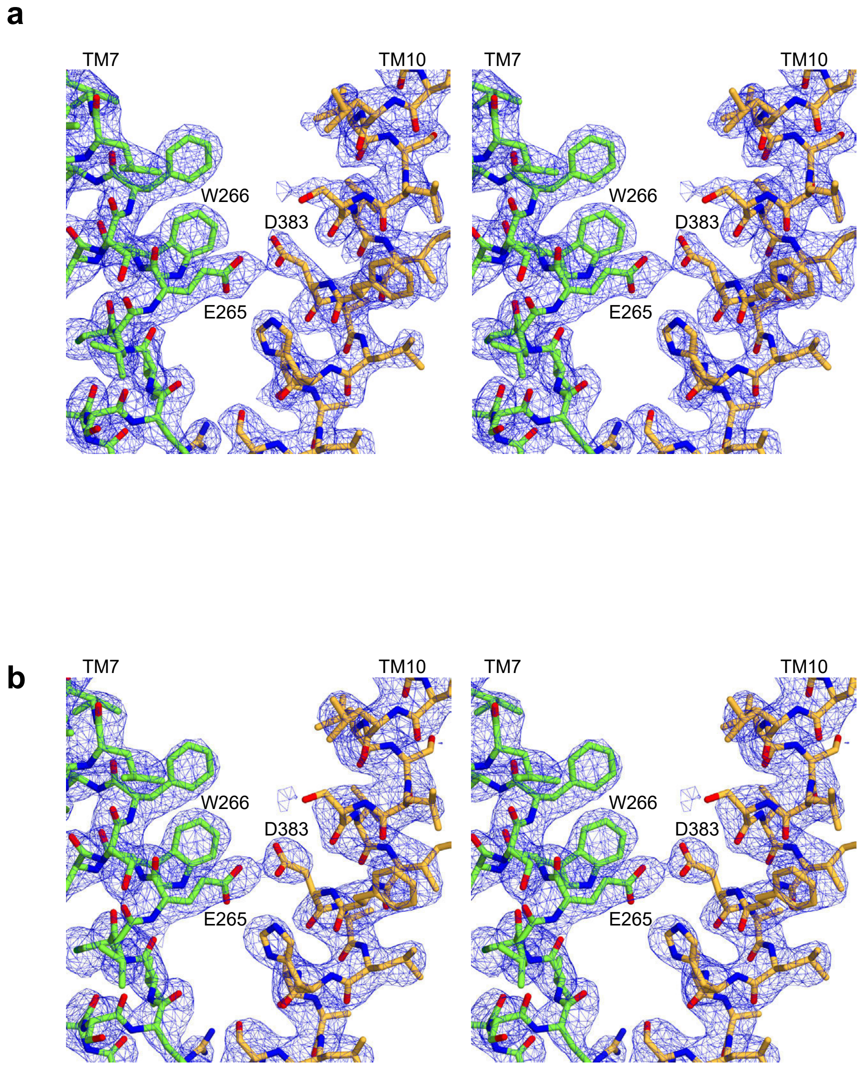

Supplementary Figure 2. The 2.6 Å electron density map of AtDTX14

(a, b) A stereo view of the 2mFo–DFc electron density map (a) and the composite omit map calculated by the program PHENIX37 (b) around TM7 and TM10, contoured at 1.0 σ.

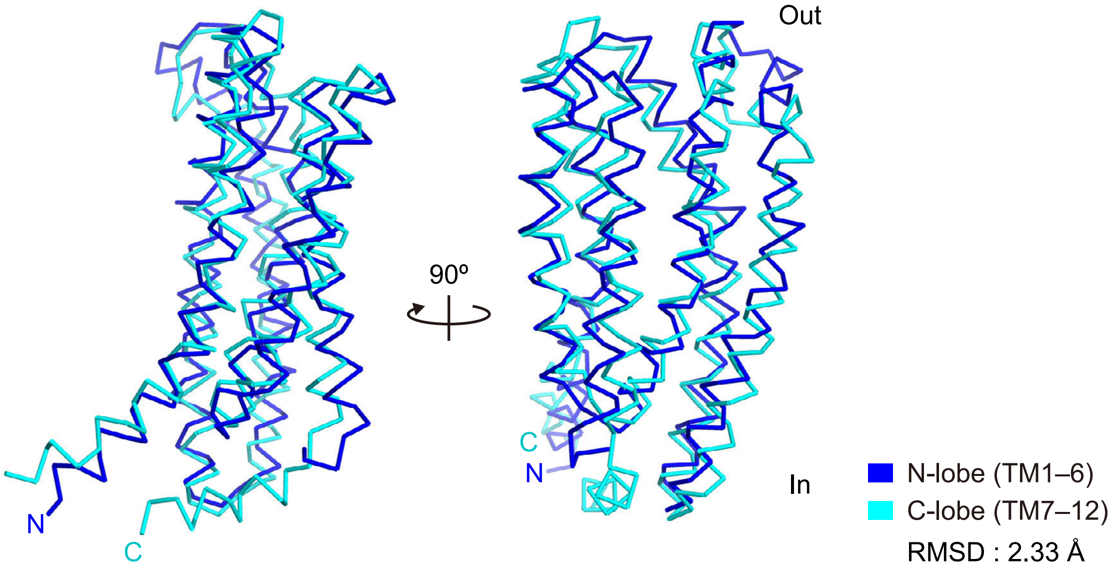

Supplementary Figure 3. Structural comparison of N- and C-lobes of AtDTX14

Superimposition of the N-lobe (blue) and the C-lobe (cyan) of AtDTX14.

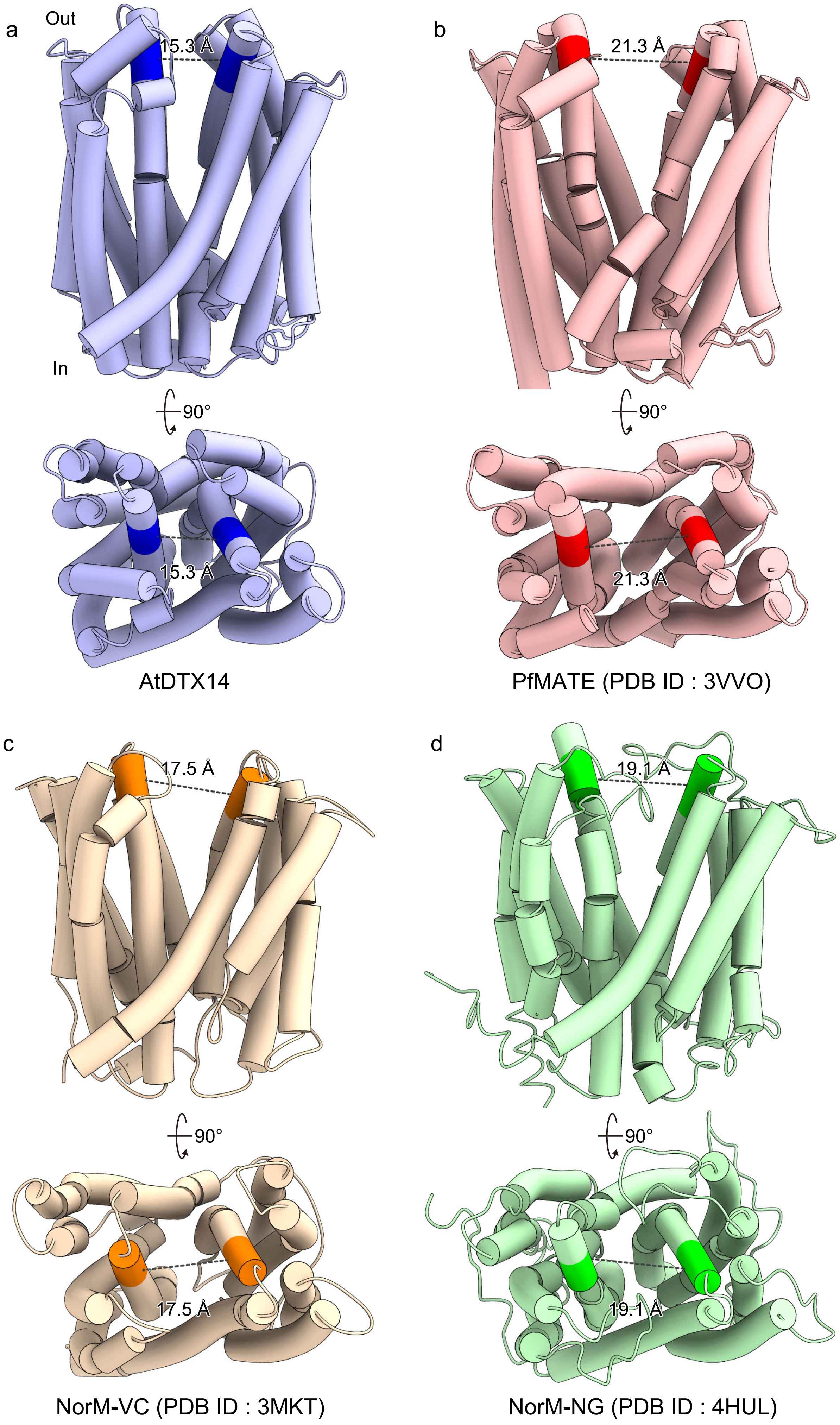
a
Out
15.3 Å
In
90°
15.3 Å
AtDTX14
b
21.3 Å
90°
21.3 Å
PfMATE (PDB ID : 3VVO)
c
17.5 Å
90°
17.5 Å
NorM-VC (PDB ID : 3MKT)
d
19.1 Å
90°
19.1 Å
NorM-NG (PDB ID : 4HUL)

Supplementary Figure 4. Comparison of the overall structures of AtDTX14, PfMATE, NorM-VC and NorM-NG

Overall structures of AtDTX14 (a), bent-conformation PfMATE (b, PDB ID: 3VVO), NorM-VC (c, PDB ID: 3MKT) and NorM-NG (d, PDB ID: 4HUL). The upper cartoons are viewed parallel to the membrane and the lower cartoons are viewed from the extracellular side. The five extracellular residues at TM2 and TM8 are highlighted, and the dashed lines represent the distances between the centers of masses of the Cα atoms in the highlighted areas.

a

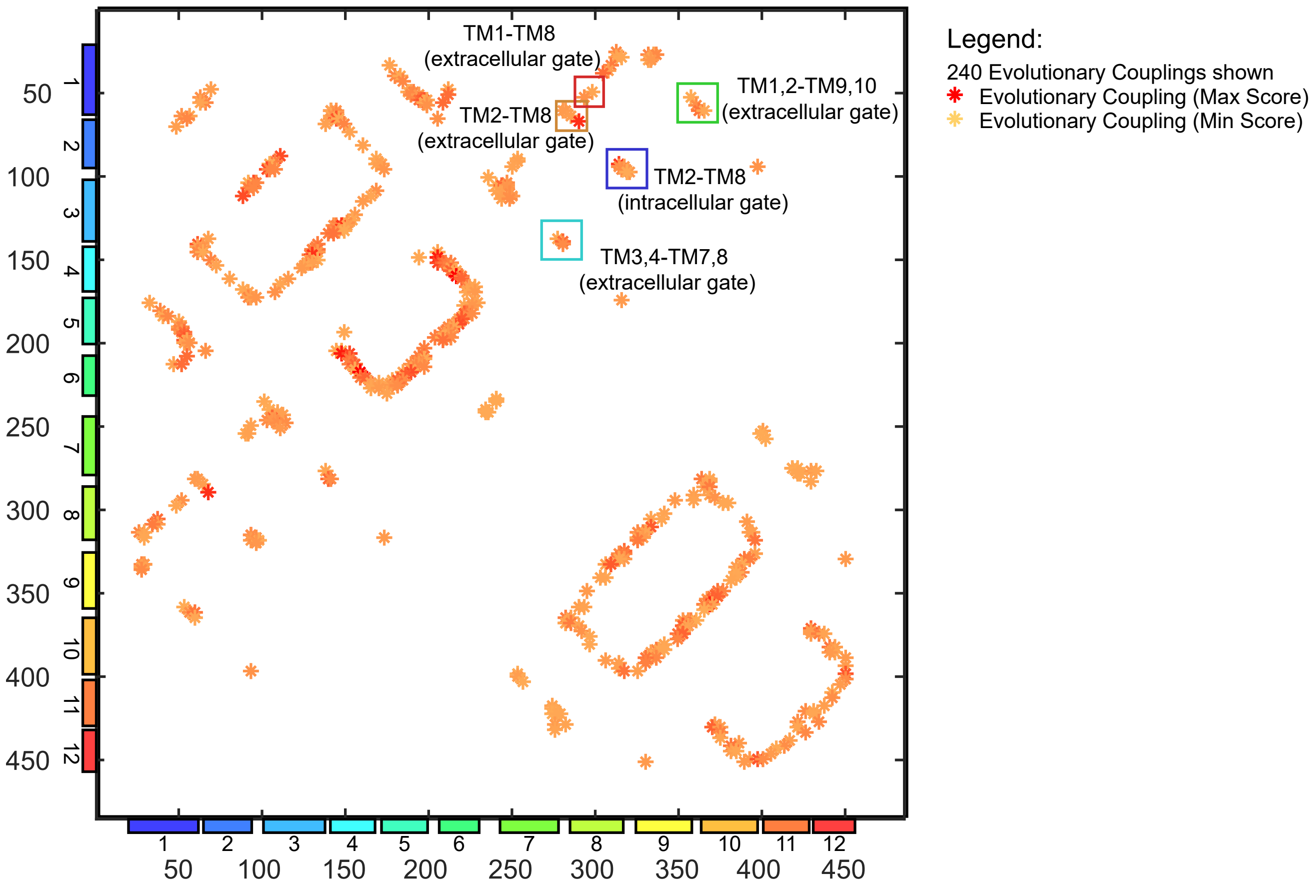

b

Extracellular gate

Residue No.

TM1	TM8	EC score
52	294	0.34940
49	298	0.23750

TM2	TM8	EC score
67	290	0.90079
61	281	0.29903
60	282	0.29865
64	285	0.28212

TM1, 2	TM9, 10	EC score
60	362	0.51245
57	361	0.36848
60	365	0.23365

TM3, 4	TM7, 8	EC score
139	281	0.57583
141	281	0.38897
138	277	0.24603

Intracellular gate

Residue No.

TM2	TM8	EC score
93	315	0.85472
96	316	0.36744
97	319	0.36053
94	315	0.35393
94	318	0.29929

c

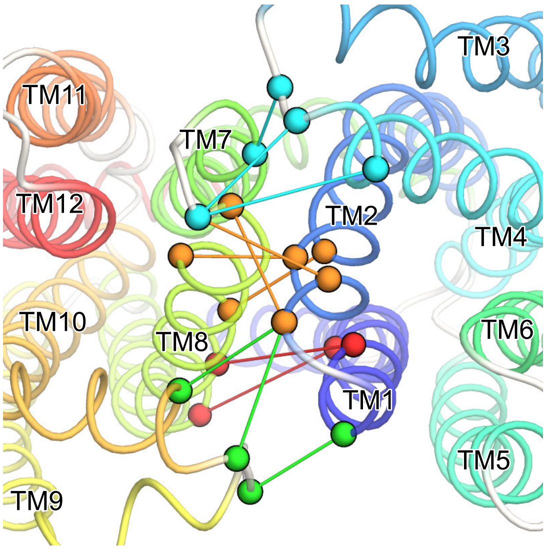

Extracellular gate (model structure)

d

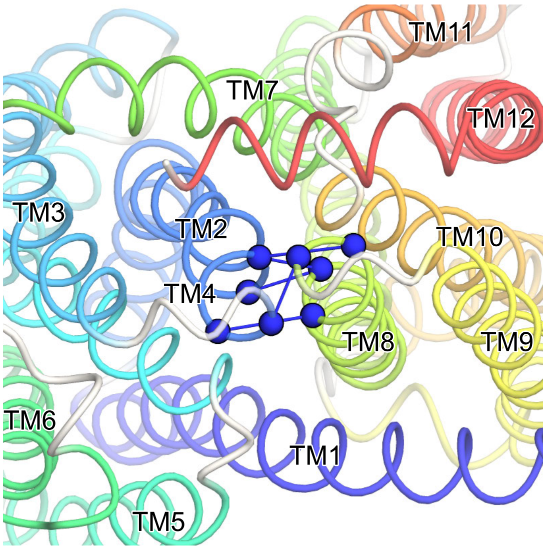

Intracellular gate (crystal structure)

Supplementary Figure 5. Evolutionary covariation analysis of MATE homologues

(a) Contact maps of the 240 top-ranked evolutionary couplings (ECs), calculated from 36,873 homologue sequences using the program EVfold_membrane. The ECs are represented as stars and colored according to the EC scores, from orange to red. The colored rectangles indicate the ECs corresponding to the putative extracellular gate interactions and the observed intracellular gate interactions. (b) The tables indicating the EC pair residues and their EC scores. The color of each table corresponds to that of the rectangles in panel (a). (c, d) Close-up views of EC residue pairs on the extracellular side (c) and the intracellular side (d). The spheres and lines indicate the EC residue pairs with high EC scores. The model structure was created based on the crystal structure of MurJ (PDB ID: 5T77). The colors of the spheres and lines correspond to those of the rectangles in panel (a).

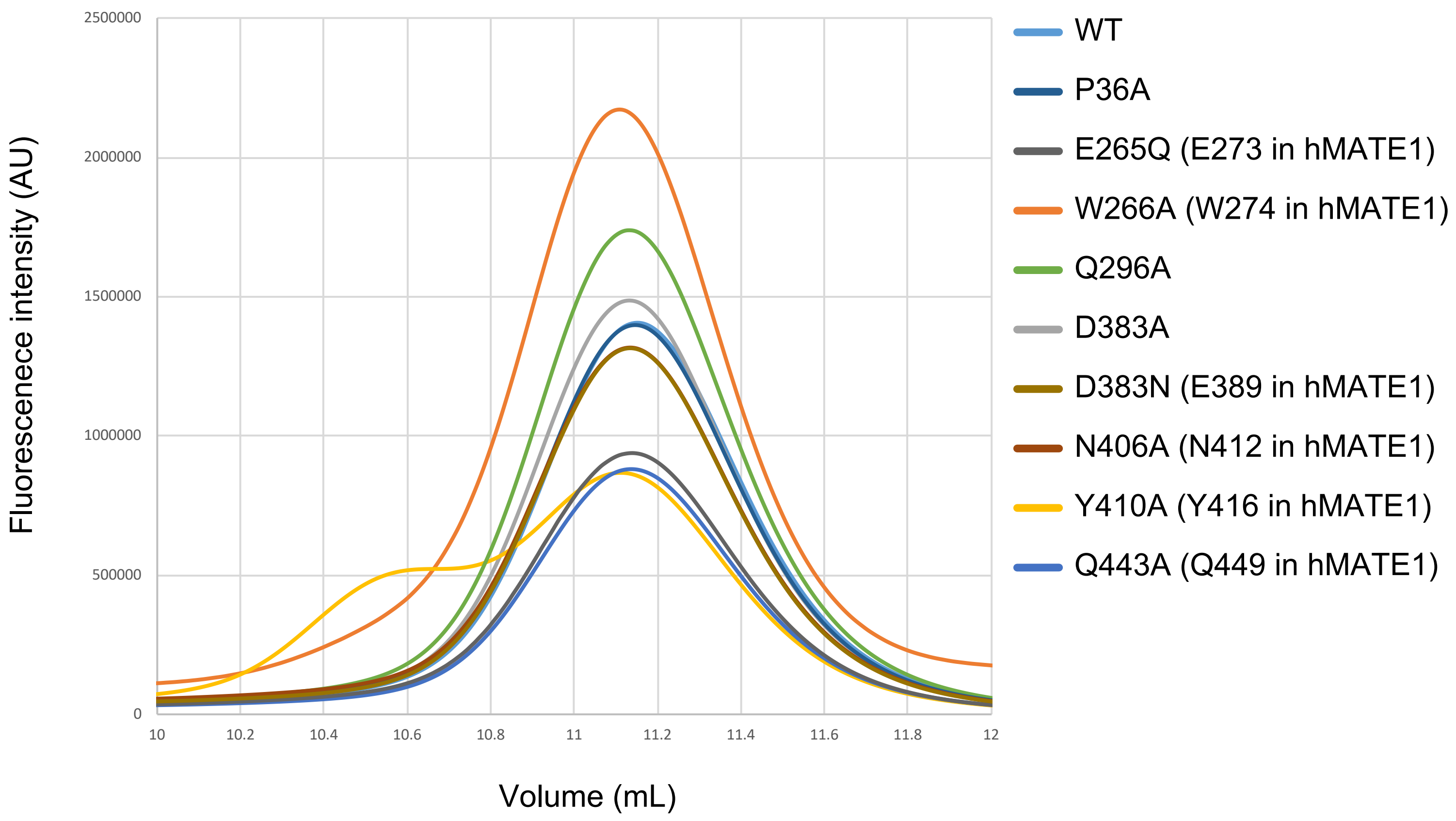

Supplementary Figure 6. Fluorescence-detection size-exclusion chromatography analyses of AtDTX14 mutants

The chromatograms of the size-exclusion chromatography analyses of the AtDTX14 mutants. All samples retained their monodispersity, indicating that all mutants maintained their structural integrity.

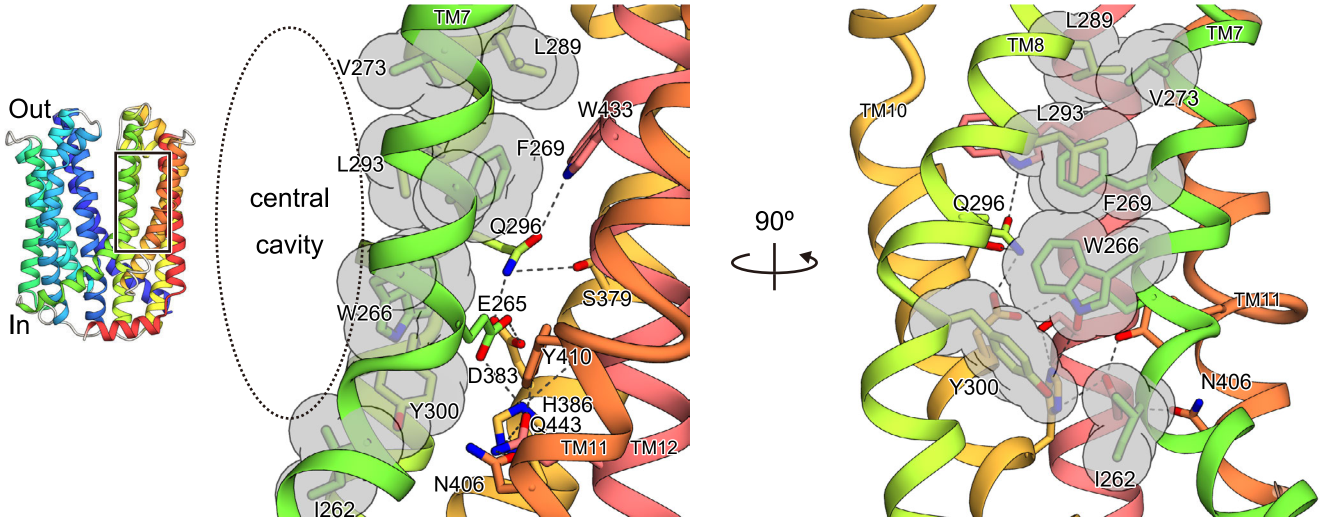

Supplementary Figure 7. Close-up views of the hydrophobic barrier

Close-up view of the hydrophobic barrier that separates the internal cavity and the hydrogen bonding network located at the C-lobe. The CPK models (gray) represent the bulky residues that construct the hydrophobic barrier.

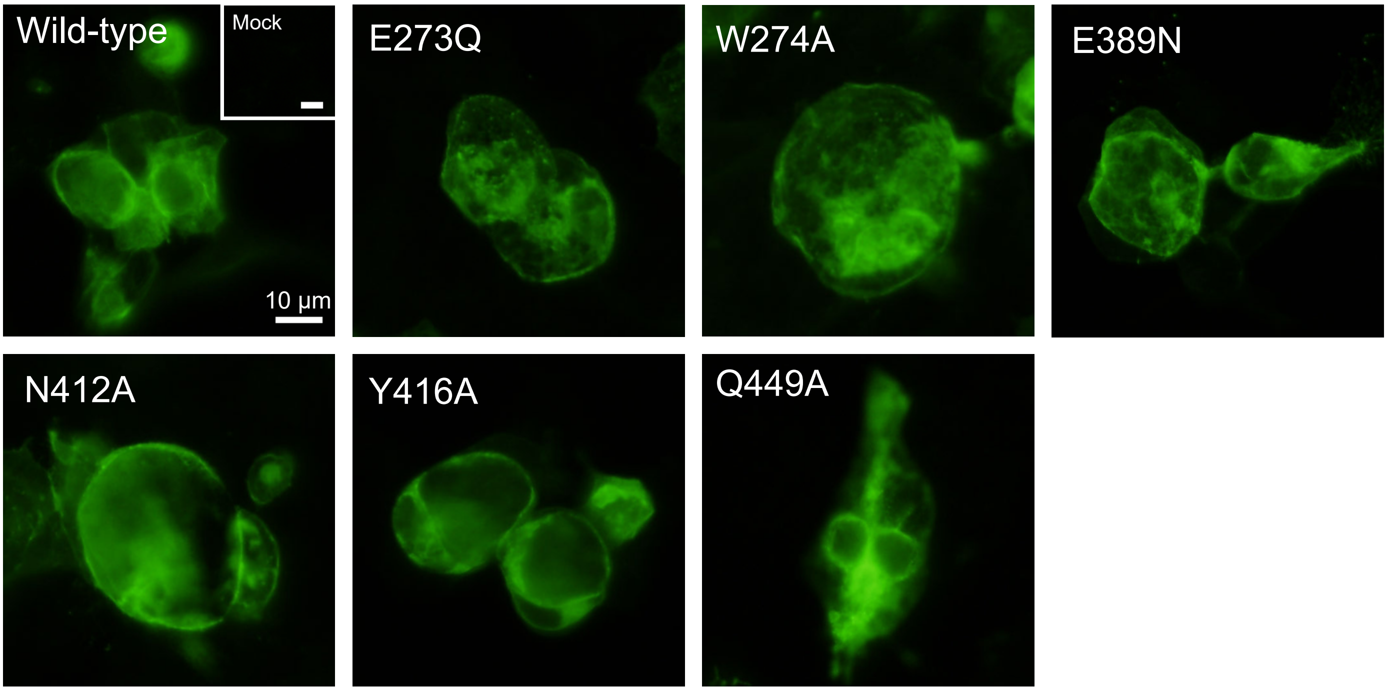

Supplementary Figure 8. Expression and localization analysis of hMATE1 mutants

Immunofluorescence analysis to examine the expression and localization of human MATE1 mutants on the cell surface membrane of HEK-293 cells. Cells were examined by indirect immunofluorescence microscopy, using an anti-hMATE1 antibody. Bar = 10 μm.

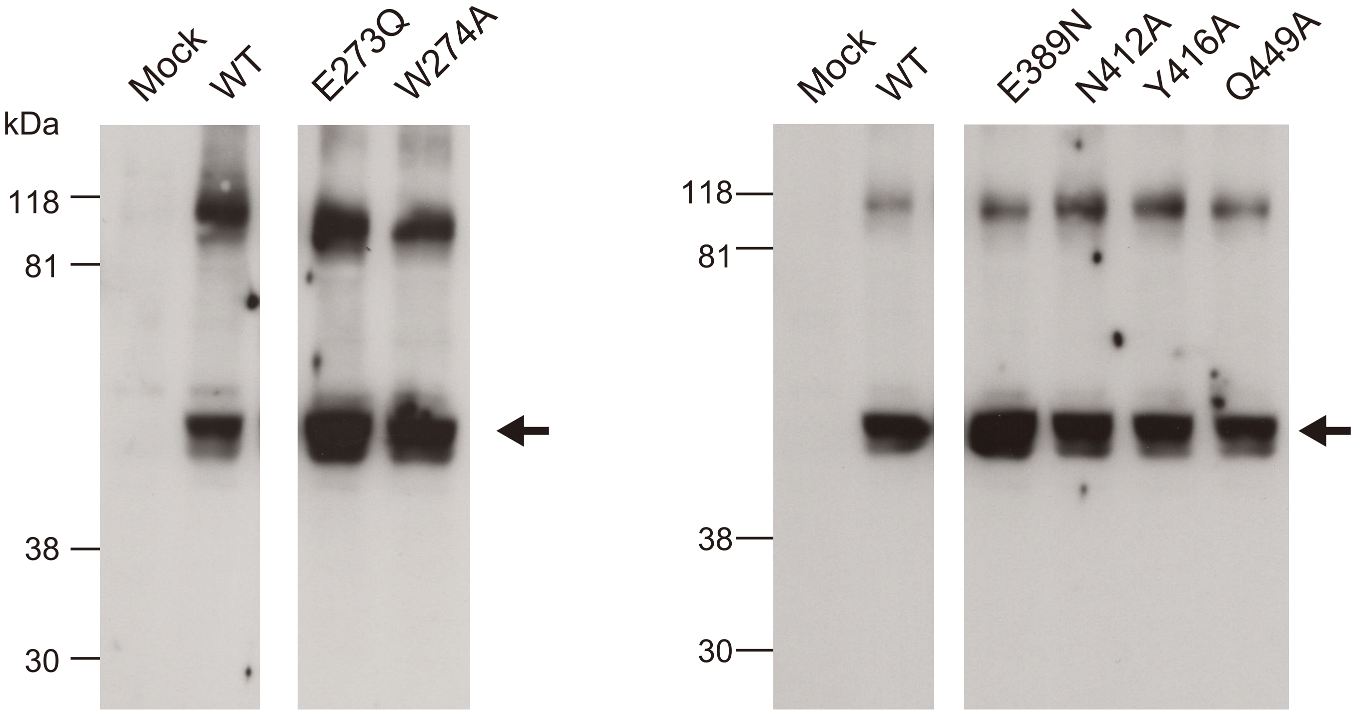

Supplementary Figure 9. Western blot analysis of hMATE1 mutants

Western blot results for representative hMATE1 mutants. The western blots probed with the anti-hMATE1 antibody confirmed the proper expression of the mutants. For the western blots, total protein extracts (20 μg) were subjected to electrophoresis. The arrows indicate the bands of hMATE1 mutants.

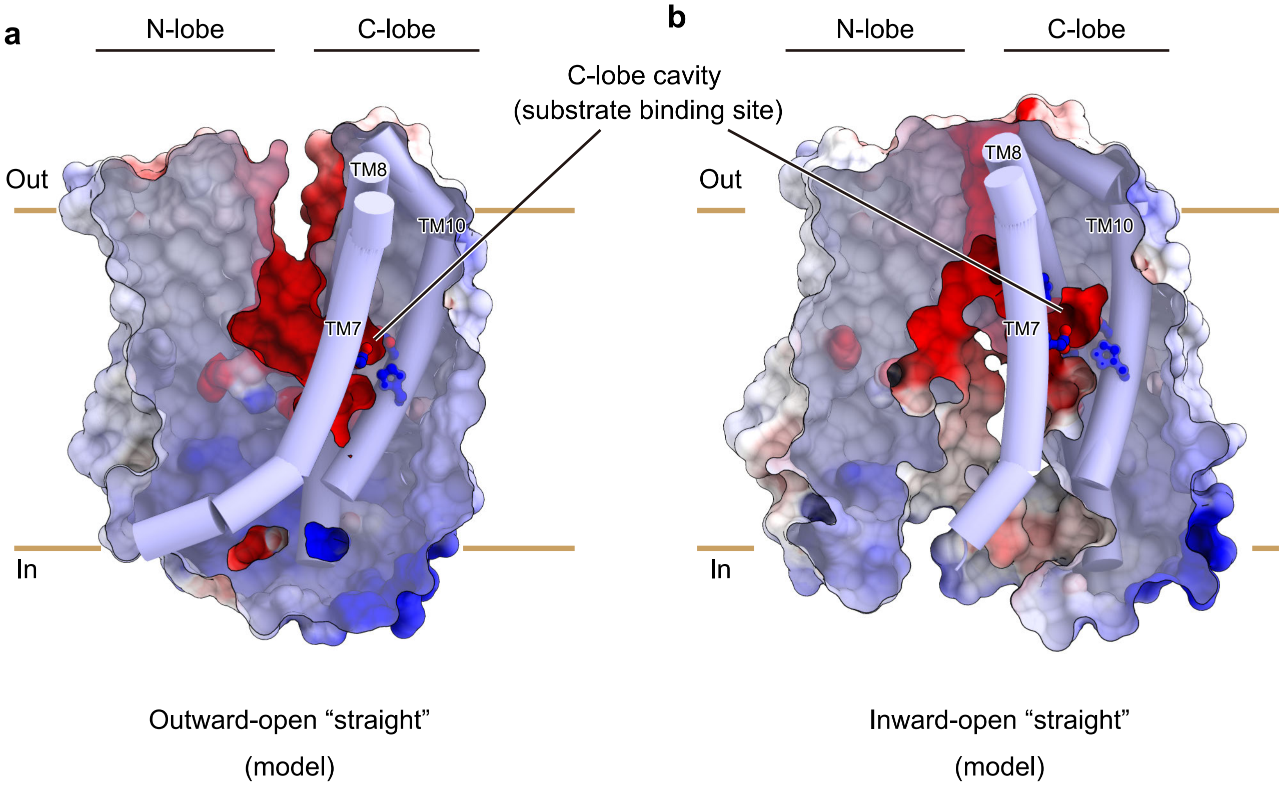

Supplementary Figure 10. Putative substrate binding site of AtDTX14

The model structures of the “TM7-straight form” of AtDTX14 in the outward-open state (a) and the inward-open state (b). The TM7-straight form of the outward-open state was created by manually adjusting the AtDTX14 structure using the program COOT[2], based on the NorM-VC structure (PDB ID : 3MKT), which is in the TM7-straight form. Then, the TM7-straight form of the inward-open state was created by respectively superimposing the N- and C-lobes of this structure onto the N- and C-lobes of the crystal structure of MurJ (PDB ID: 5T77), using the SSM superpose algorithm[3]. The surface models are colored according to the electrostatic potential from red (–10 kbT/e) to blue (+10 kbT/e).

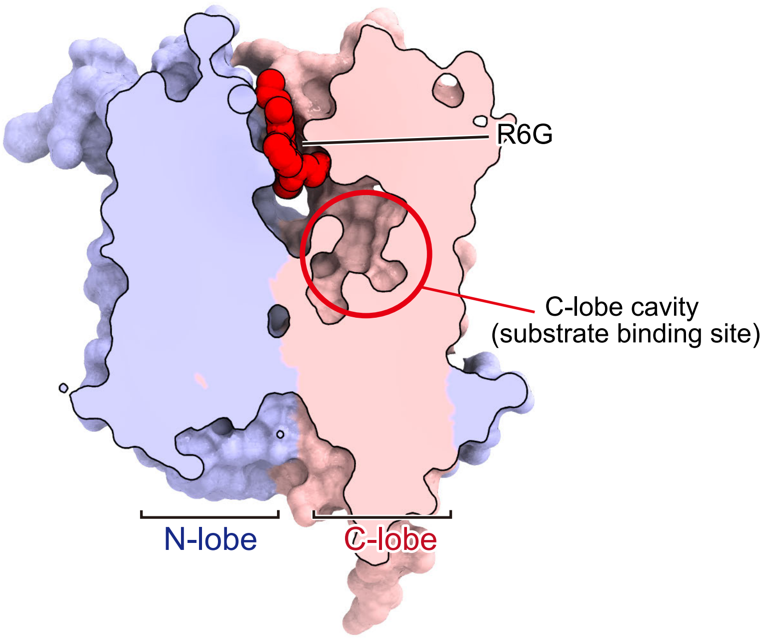

4HUN: NorM-NG with R6G

Supplementary Figure 11. Substrate binding site of NorM-NG

Surface representation of the crystal structure of NorM-NG in complex with R6G (PDB ID: 4HUN). The red colored CPK model indicates the R6G molecule.

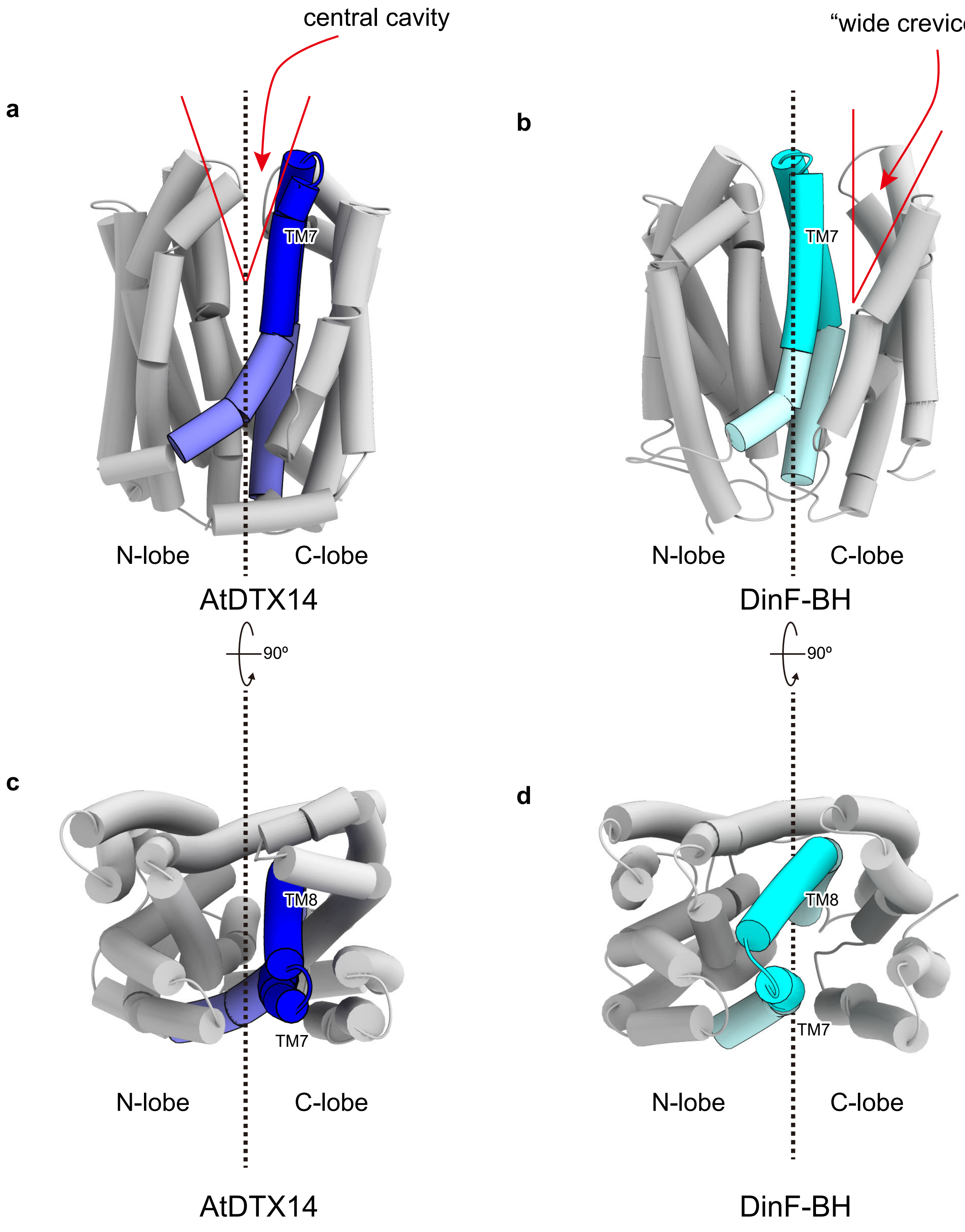

Supplementary Figure 12. Structural comparison between AtDTX14 and DinF-BH

Cartoon representations of AtDTX14 and DinF-BH (PDB ID: 4LZ6) viewed parallel to the membrane (a, b) and perpendicular to the membrane (c, d). TM7 and TM8 are highlighted. Red arrows indicate the "central cavity" or the "wide crevice" respectively, which are the paths for substrates in each model.

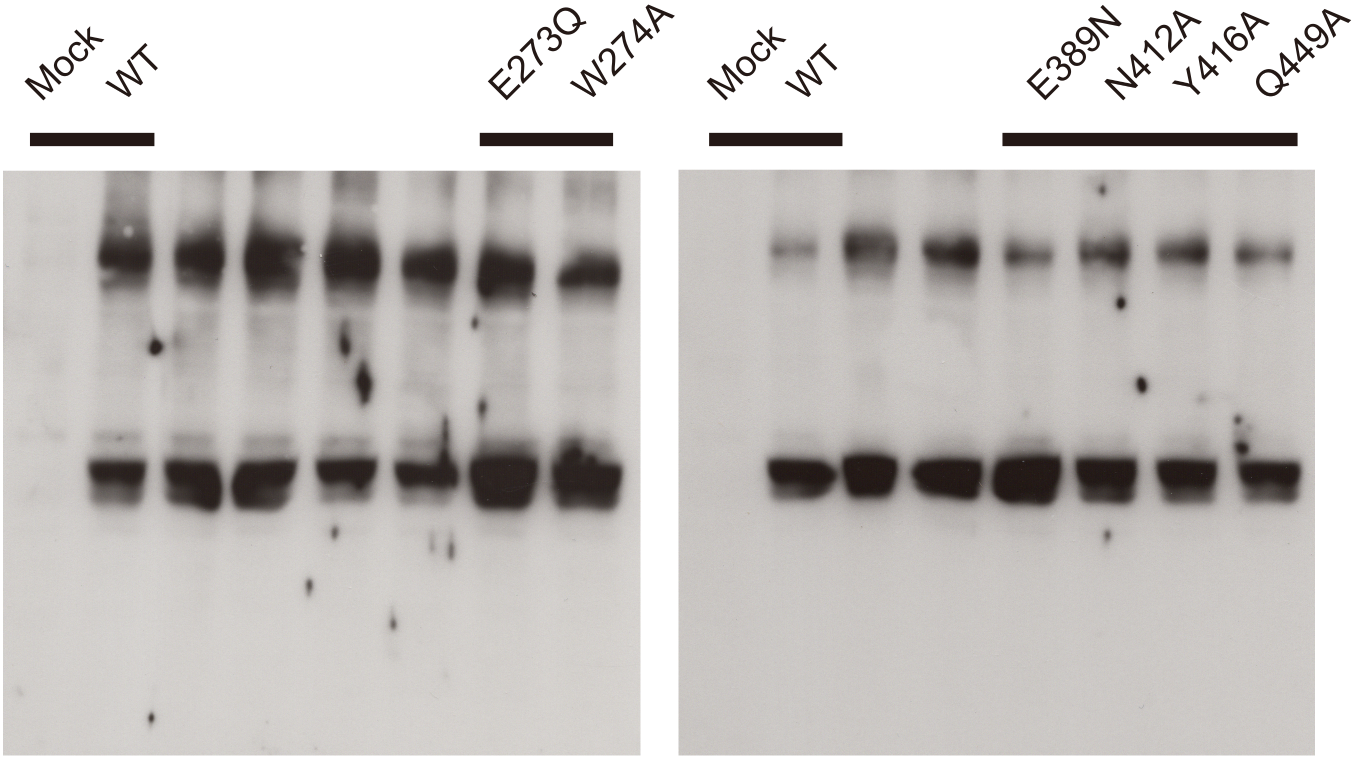

Supplementary Figure 13. Uncropped images of the blots shown in the Supplementary Figure 9

Supplementary Table 1. The nucleotide sequence of the synthesized AtDTX14 and the crystallization construct of AtDTX14 used in this study

$AtDTX14_{WT}$	ATGGACTCGGCGGAAAAAGGCTTGCTCGTCGTTAGTGATAGGGAGGAAGTGAACAAAAAGGATG<br>GATTCTTGCGTGAGACCAAAAAGCTGAGCTACATCGCTGGTCCCATGATTGCCGTGAACTCCAGCA<br>TGTACGTGCTGCAGGTCATCTCTATTATGATGGTCGGCCACCTCGGAGAACTCTTCTTGTCTTCAAC<br>AGCCATCGCAGTGTCGTTCTGCTCCGTCACGGGTTTCAGCGTGGTCTTCGGCCTGGCGTCTGCTCTG<br>GAAACACTCTGTGGTCAGGCCAACGGCGCAAAGCAATACGAGAAATTGGGAGTGCATACCTACAC<br>TGGTATCGTTAGCCTGTTCCTCGTGTGCATTCCACTGTCTCTGCTCTGGACGTACATCGGTGACATT<br>TTGTCCCTGATCGGCCAGGATGCGATGGTGGCTCAAGAGGCCGGCAAGTTCGCTACCTGGCTGATT<br>CCAGCACTCTTCGGCTACGCGACTTTGCAGCCGCTGGTCCGCTTCTTCCAGGCCCAAAGCCTCATC<br>TTGCCTCTGGTCATGAGTTCGGTCTCCAGCCTGTGCATCCACATTGTCTTGTGTTGGTCACTGGTTT<br>TCAAGTTCGGTCTCGGCAGTTTGGGCGCTGCCATCGCTATTGGAGTGTCTTACTGGCTCAACGTCA<br>CCGTTCTCGGATTGTACATGACTTTCTCTTCAAGTTGTTCAAAAAGTCGCGCCACCATCTCGATGTC<br>CCTGTTCGAAGGAATGGGAGAGTTCTTCCGTTTCGGTATCCCAAGCGCTTCTATGATTTGCCTCGA<br>ATGGTGGTCATTCGAGTTCTTGGTCTTGCTGAGTGGCATCCTCCCTAACCCCAAGTTGGAAGCCAG<br>CGTTCTCTCTGTGTGTTTGTCCACACAGTCGTCCTTGTACCAAATCCCTGAATCACTGGGCGCAGCG<br>GCTAGTACGAGGGTGGCTAACGAGCTGGGCGCCGGAAACCCCAAACAGGCAAGAATGGCGGTCT<br>ACACAGCTATGGTTATCACGGGAGTGGAGTCAATCATGGTCGGCGCAATTGTTTTCGGAGCCAGG<br>AACGTGTTCGGTTACCTCTTCAGCTCTGAAACTGAGGTTGTGGACTACGTGAAGTCCATGGCTCCA<br>CTCTTGTCATTGAGTGTCATCTTCGATGCTCTGCACGCAGCACTCTCGGGAGTTGCTAGAGGTTCCG<br>GACGTCAAGACATCGGAGCTTACGTGAACCTGGCGGCTTACTACCTCTTCGGAATCCCGACAGCA<br>ATTCTGCTCGCGTTCGGTTTCAAGATGCGCGGACGTGGTCTGTGGATCGGAATTACCGTCGGTAGC<br>TGCGTCCAGGCCGTTTTGCTGGGCTTGATCGTTATTCTGACTAACTGGAAGAAACAAGCTCGCAAG<br>GCCAGGGAAAGAGTCATGGGAGATGAATACGAGGAAAAAGAGTCGGAGGAAGAGCATGAGTACA<br>TCTCC
$AtDTX14_{cryst}$	AAGGATGGATTCTTGCGTGAGACCAAAAAGCTGAGCTACATCGCTGGTGCCATGATTGCCGTGAAC<br>TCCAGCATGTACGTGCTGCAGGTCATCTCTATTATGATGGTCGGCCACCTCGGAGAACTCTTCTTGT<br>CTTCAACAGCCATCGCAGTGTCGTTCTGCTCCGTCACGGGTTTCAGCGTGGTCTTCGGCCTGGCGTC<br>TGCTCTGGAAACACTCTGTGGTCAGGCCAACGGCGCAAAGCAATACGAGAAATTGGGAGTGCATA<br>CCTACACTGGTATCGTTAGCCTGTTCCTCGTGTGCATTCCACTGTCTCTGCTCTGGACGTACATCGGT<br>GACATTTTGTCCCTGATCGGCCAGGATGCGATGGTGGCTCAAGAGGCCGGCAAGTTCGCTACCTGG<br>CTGATTCCAGCACTCTTCGGCTACGCGACTTTGCAGCCGCTGGTCCGCTTCTTCCAGGCCCAAAGCC<br>TCATCTTGCCTCTGGTCATGAGTTCGGTCTCCAGCCTGTGCATCCACATTGTCTTGTGTTGGTCACTG<br>GTTTTCAAGTTCGGTCTCGGCAGTTTGGGCGCTGCCATCGCTATTGGAGTGTCTTACTGGCTCAACG<br>TCACCGTTCTCGGATTGTACATGACTTTCTCTTCAAGTTGTTCAAAAAGTCGCGCCACCATCTCGAT<br>GTCCCTGTTCGAAGGAATGGGAGAGTTCTTCCGTTTCGGTATCCCAAGCGCTTCTATGATTTGCCTC<br>GAATGGTGGTCATTCGAGTTCTTGGTCTTGCTGAGTGGCATCCTCCCTAACCCCAAGTTGGAAGCCA<br>GCGTTCTCTCTGTGTGTTTGTCCACACAGTCGTCCTTGTACCAAATCCCTGAATCACTGGGCGCAGC<br>GGCTAGTACGAGGGTGGCTAACGAGCTGGGCGCCGGAAACCCCAAACAGGCAAGAATGGCGGTCT<br>ACACAGCTATGGTTATCACGGGAGTGGAGTCAATCATGGTCGGCGCAATTGTTTTCGGAGCCAGGA<br>ACGTGTTCGGTTACCTCTTCAGCTCTGAAACTGAGGTTGTGGACTACGTGAAGTCCATGGCTCCACT<br>CTTGTCATTGAGTGTCATCTTCGATGCTCTGCACGCAGCACTCTCGGGAGTTGCTAGAGGTTCCGGA<br>CGTCAAGACATCGGAGCTTACGTGAACCTGGCGGCTTACTACCTCTTCGGAATCCCGACAGCAATT<br>CTGCTCGCGTTCGGTTTCAAGATGCGCGGACGTGGTCTGTGGATCGGAATTACCGTCGGTAGCTGC<br>GTCCAGGCCGTTTTGCTGGGCTTGATCGTTATTCTGACTAACTGGAAGAAACAAGCTCGCAAGGCC<br>AGGGAAAGAGTCATGGGAGATGAATAC

Supplementary Information References

1. Kumar, S. et al. MEGA7: Molecular Evolutionary Genetics Analysis Version 7.0 for Bigger Datasets. Mol. Biol. Evol. 33, 1870–1874 (2016).

2. Emsley, P., Lohkamp, B., Scott, W. G. & Cowtan, K. Features and development of Coot. Acta Cryst 66, 486–501 (2010).

3. Krissinel, E. et al. Secondary-structure matching (SSM), a new tool for fast protein structure alignment in three dimensions. Acta Crystallogr. Sect. D Biol. Crystallogr. 60, 2256–2268 (2004).
